# Fusospirillary Peridental Gingivitis*This is an abstract of a paper read before the Royal Society of Medicine, as printed in the *Dental Record* for March, 1917.

**Published:** 1917-05

**Authors:** Frank E. Taylor, W. H. M’Kinstry

**Affiliations:** Captain R.A.M.C.


### F[illegible]SOSPIRILLARY PERIDENTAL GINGIVITIS *

BY FRANK E. TAYLOR, M.D., and W. H. M'KINSTRY, M.B., CAPTAIN R.A.M.C.

It is to the condition of ulcerative fusospirillary peridental gingivitis and its relationship to Vincent's angina of the pharynx and to the character of the organisms found in the two conditions, that we desire to call attention in this communication.

On examination of the gums and teeth in patients the subjects of this infection usually reveal the following conditions: The teeth are frequently tartarated and unclean, showing evidence of great neglect—although this is not invariably the case, as in many of our patients the sound and well-groomed condition of the teeth was particularly noticeable. In an advanced and well-marked case the

* This is an abstract of a paper read before the Royal Society of Medicine, as printed in the *Dental Record* for March, 1917.

gums are red, swollen and ulcerated in immediate contact with the neck of the teeth, and extending out for a variable distance seldom exceeding one-fourth of an inch. The ulcerated surface is often covered with a soft, gray, foul-smelling exudation. The slightest touch with a swab or platinum loop to the ulcerated surface causes profuse bleeding. Any attempt on the patient's part to use a toothbrush also causes free bleeding, so that it must be temporarily discarded. The extent of the ulceration varies greatly, being limited in some cases to the neck of one or two teeth, in others extending the whole length of the upper or lower gums. The infection seems to show predilection for certain situations, being most frequently found about the lower central incisors, and behind the last lower molars. It is also frequently found about crowned and stopped teeth. In some cases the ulceration is limited to the tops of the papillæ between the teeth and to the parts of the gum separated from the teeth. In the slighter cases the ulceration around the necks of the teeth may not be readily visible, though the patient may only complain of bleeding of the gums on cleaning the teeth with a toothbrush. In these cases the affected areas are readily found and exposed by lightly rubbing the gums with a pledget of cotton wool, when oozing of blood immediately occurs. In the more severe cases, in addition to the bleeding, the patients complain of tenderness on pressure and on mastication; whilst in two or three very long standing cases the affected teeth have become quite loose. In the majority of our cases the ulceration has developed slowly, and been of long standing, the patients stating that the gums have bled readily, and a foul taste has been present in the mouth for many weeks, months, or even years, and it is in these latter cases that the loosening of the teeth has been observed. In a few cases, on the other hand, the disease seems to run a more acute course, extensive ulceration developing in a few days. Hence the cases can be classified as acute or chronic. However developed, whether rapidly or slowly, the same condition results. The temperature in the uncomplicated disease is never raised, nor are the lymphatic glands enlarged or tender.

With regard to the etiology of this affection, its widespread prevalence amongst the troops in London at the present time suggests its infectivity, which is definitely proved by the results of bacteriological examination.

Only the other day, and after this paper was written, the following suggestive history, indicating how the infection might be spread by toothbrushes, was brought to our notice. Sixteen men in billets in a certain private house were in the habit of keeping their toothbrushes on a common shelf. We have already had three of these sixteen men for treatment, and have examined the toothbrushes of two of them with positive results. About four weeks ago the third patient accidentally used one of the brushes since found to be infected, and to this he attributes his complaint, the gums becoming sore and bleeding about one week later.

The diagnosis of the disease is a comparatively simple matter. The character of the ulceration of the gum in close proximity to the neck of the teeth, with special predilection for the lower central incisors and wisdom teeth, the overlying soft grayish exudation, the readiness with which bleeding is elicited, and the fœtor of the breath, all provide a clinical picture by which the disease can readily be recognized. This diagnosis is readily confirmed by a bacteriological examination, the microscope revealing the presence of fusiform bacilli and spirilla—both Gram-negative organisms, either alone or together with a variety of other organisms—cocci, bacilli and leptothrices.

Many of our patients had already been under treatment, often for a considerable time, usually without much improvement, if any at all. They came to us with a diagnosis of pyorrhea already made, and in some cases had been advised to have their teeth extracted. Now in this condition oozing of pus from the tooth sockets, and the presence of pockets of pus, as seen in pyorrhea, do not occur, and we consider that this disease should be sharply differentiated from pyorrhea. The clinical history, the treatment and the prognosis of the two conditions are totally different.

As regards treatment, this is not commenced until the diagnosis has been established by bacteriological examination. Only those cases in which the typical Gram-negative fusiform bacilli and spirilla are found are included under this heading. In those cases in which the teeth are unclean and tartarated, the patient is sent to the dental department, where the teeth are scaled, and the tartar and membranous deposit are removed from the gums with a glycothymoline spray, under a pressure of 30 to 40 pounds to the square inch. The gums are then carefully dried with pledgets of absorbent wool, and afterwards painted freely with 0.2 per cent. alkaline solution of salvarsan or kharsivan as prepared for intravenous injection. The drying and painting of the gums are then repeated at least once, and preferably twice, daily. On account of the tenderness of the gums, the scaling of the teeth may need to be done at two or three sittings, the swabbing of the gums with salvarsan being carried out between the sittings. Usually marked improvement is noticed in twenty-four to forty-eight hours, and the disease yields entirely to treatment in the majority of the cases within fourteen days if daily applications have been thoroughly and systemically carried out. In a few cases where improvement appears to be somewhat delayed under this treatment, we vary the medicament which is applied locally, the most successful results being obtained with a mixture containing vinum ipecacuanhæ, liquor arsenicalis and glycerin.

DISCUSSION

Mr. Percy Edgelow: I am much interested in Dr. Taylor's and Captain McKinstry's paper, having attended a considerable number of officers of the Royal Flying Corps for acute septic gingivitis. I had entertained an impression that the particular nature of their avocation was in some way responsible for the prevalence of their trouble, but after the author's remarks as to the frequency with which he has come across it in other branches of the Army, it is obvious that the particular conditions surrounding "flying" will not suffice to explain these attacks. I agree with him that this gingivitis is an affection distinct from pyorrhea alveolaris, though no doubt they are to be found coexistent in the same mouth. I also have found that the gums and soft tissues around the wisdom teeth are very liable to be affected, particularly in the lower jaw, with accompanying swelling and tenderness of the neighboring pharyngeal mucous membrane, a painful submaxillary gland being often present. The adjacent mucous membrane of the cheek is occasionally so swollen as to intrude between the opposing molars and is lacerated when biting, causing a very painful, inflamed and ulcerated condition and leading to difficulty in opening the mouth beyond one-third of its normal width. The fœtor of the breath is great and there is often pronounced general malaise. The swollen condition of the gums leads to free bleeding on the slightest instrumentation, and to the formation of pockets between the necks of the teeth, particularly in the molar and premolar regions. Marginal sloughs are frequently present. Solid food is avoided in very acute cases on account of the pain during biting, and fluid nourishment alone is tolerated. The affection is apparently uninfluenced by the actual condition of the teeth as regards caries, many of the acutest attacks coming under my notice having occurred in mouths possessing a full and sound set of teeth. As regards treatment, I first give an iodine wash to rinse the mouth thoroughly and then freely apply a mixture of equal parts of carbolic acid and camphor. The camphor prevents the caustic and irritating effect of carbolic acid used in a strength of 1 in 2. The extreme tenderness and free bleeding quickly yield to this treatment. For home use I rely on exceptionally severe cases with much swelling and ulceration of the mucous membrane near the wisdom teeth accompanied by laceration of the cheek mucous membranes. I have extracted the upper third molar tooth under novocaine with rapidly beneficial results. I select the upper tooth for three reasons: (1) It articulates with the lower wisdom tooth alone, whilst the latter has the benefit of articulating with both the second and third upper molars; (2) it is much easier to extract than the lower wisdom; (3) owing to its better drainage the upper wound heals more rapidly than the lower one.

Mr. Schelling: Are cultivation and other means requiring considerable time necessary to confirm the existence of the microorganisms described? For the cases which I have been called upon to treat presenting a similar appearance to those just described are generally those of officers, home on six or ten days' leave, who are anxious to have as much relief as possible in the fewest number of visits. The treatment adopted consists of, at first visit: (1) Free swabbing with peroxide of hydrogen 10 vols.; (2) complete painting with lin. iodi. applied on a small pellet of cotton and used both for its antiseptic action and its property of staining all the tartar so that it can be more easily removed with appropriate fine scalers; (3) polishing with a buff and pumice powder; (4) seeking for pockets and packing them with cotton plugs moistened in saturated solution of chinosol, the pockets behind the wisdom teeth being similarly packed with small plugs moistened in pure carbolic acid. A second visit next day to remove the plugs and complete the scaling and apply iodine, and, if necessary, more visits for chinosol dressings in pockets on each occasion, and, finally, instructions to the patient thoroughly to brush the gums inside and out daily, with a palate brush and a little table salt and to return for inspection at the next opportunity.

Mr. A. Parker Cater (Lieutenant G.B.C., 36 Corps d'Armée française): One of many experiences in connection with the work and organization of the dental operating vans which I took to France, for service with the French army at the front, has been the large and increasing number of cases of what we called ''trench gingivitis'' and known on ''this side'' as ''trench gum.'' Whatever may be its correct nomenclature, the fact remains that its lowering effects on the men's general physical condition and efficiency is most marked. Not only does it attack the gums around the teeth, but it also shows itself in patches of varying size over the gums and inside the lips and cheeks according to the severity of the case. At a certain stage it might have been mistaken for ''pyorrhea'' pure and simple, but the extreme rapidity with which the symptoms developed appeared to me to suggest a specific organism as being the actual cause of the complaint. The ''services'' of these motor dental surgeries being chiefly devoted to men *coming out of* trenches, led one at first to think that the trouble might possibly be confined to the front line troops. I am now, however, constantly discovering that the complaint is spreading, not only to men who have never been near the trenches or even out of England, but also to civilians. In practically all these cases it appears to be pretty certain that they have been in actual or indirect contact with infected individuals. It has been suggested to me that it is invariably caused by neglect or want of cleanliness. As against this, and also to demonstrate how highly infectious it appears to be, I have frequently found it in mouths where there was no lack of cleanliness. As I have had attacks myself, one may fairly anticipate that this would not be brought about by any neglect on my own part. As already stated, I am of opinion that some special organism is the active cause of the trouble. It is highly infectious and need not necessarily be caused by lack of or inability to secure "oral" cleanliness, because if such lack of cleanliness was the dominating cause of the trouble, one might expect, practically, nearly every man at the front to be affected. The treatment indicated, as far as my experience goes, appears to be thorough scaling and cleaning as soon as the tenderness permits; frequent irrigation under pressure; applications of liquor arsenicalis with vini ipecac and glycerin alternated with salvarsan, also iodin; frequent and *proper* use of *clean* toothbrushes and vibratory or digital massage. In stubborn cases I found that ionic medication yielded quick and satisfactory results. Educational treatment consists in teaching men to avoid using each other's drinking bottles or other utensils, which in spite of army regulations they persist in doing; the passing on of pipes, cigarettes, towels, etc. The renewal of toothbrushes should also be more frequent. As far as possible a rapid examination of troops leaving trenches for rest camps has been made, and on several occasions I have found that a man with a comparatively clean mouth, would, in two or three days, develop an acute condition of trench gum, with putrescent discharge around necks of teeth, bleeding and swollen gums, teeth loose and so tender as to be useless for masticating; fetid breath, furred tongue and general feeling of "malaise" and slackness. If a man develops this acute condition in the trenches, which he frequently does, he becomes of little practical use as a fighting unit, he can not tackle his trench rations, loses nerve and also becomes an infectious menace to his fellows. The "toilette" of the mouth whilst in the trenches is very difficult, and often impossible, unless men can be provided, as part of their kit, with a paste in the form of kolynos, or some such preparation—which they are not.

As a great many civilian, as well as military, dental surgeons are now constantly having to deal with this complaint, it will be very helpful and interesting to obtain their experiences and opinions on the subject. At one base consisting mainly of chefs and cooks engaged in breadmaking, we recorded no cases of trench gingivitis. There were nearly 700 of these cooks. I have not found any cases of the complaint in men suffering from *fractures* or *traumatic* injuries of the mouth, either in France or on this side.

Dr. J. D. Rolleston: Is not the condition described by Dr. Taylor and Captain McKinstry the same as that described by Vincent as "ulceromembranous stomatitis," which is due to the same organisms as Vincent's angina, and was occasionally associated with it? Being attached to the Val de Grâce military hospital, Vincent especially noted its occurrence amongst soldiers and its liability to arise in connection with the last molar tooth. It was frequently seen among the recruits from Normandy and Savoy whose teeth were predisposed to caries by drinking cider. As medical officer in a fever hospital I have been interested in the condition for many years. Like Vincent's angina, it may occur as a primary condition or be secondary to some other acute infection, such as scarlet fever or measles. I agree with what the authors have said as to the absence or slight degree of general disturbance. I lay special stress on the peculiar fœtor as of diagnostic value. As regards what has been said about the occurrence of fusiform bacilli in suppurative conditions, it may be noted that Vincent found them in a number of suppurative processes in connection with the alimentary tract as well as in a case of pyothorax. What relation do the authors think this condition bears to cancrum oris, which I regard as a further stage of ulceromembranous stomatitis, just as gangrenous angina is a further stage of Vincent's angina? In all these conditions the fusospirillary symbiosis is met with. As regards prognosis and treatment

I have found that the condition as a rule is mild and yields readily to simple treatment, such as applications of tincture of iodin, methylene blue, or a mouth wash of potassium chlorate and myrrh. In rare cases local application of salvarsan may be required.

At the conclusion of the discussion the authors accepted the president's suggestion that the word "gingivitis" should be used instead of "periodontitis."

